# Frailty, markers of immune activation and oxidative stress in HIV infected elderly

**DOI:** 10.1371/journal.pone.0230339

**Published:** 2020-03-18

**Authors:** Susana Álvarez, Fátima Brañas, Matilde Sánchez-Conde, Santiago Moreno, Juan Carlos López-Bernaldo de Quirós, Mª Ángeles Muñoz-Fernández

**Affiliations:** 1 Laboratorio Inmuno-Biología Molecular (LIBM), Immunology Section, Hospital General Universitario Gregorio Marañón, Madrid, Spain; 2 Instituto de Investigación Sanitaria Gregorio Marañón (IISGM), Madrid, Spain; 3 Geriatrics Department, Hospital Universitario Infanta Leonor, Madrid, Spain; 4 Infectious Diseases Department, Hospital Universitario Ramón y Cajal, Madrid, Spain; 5 Instituto de Investigación Ramón y Cajal (IRyCIS), Madrid, Spain; 6 HIV Unit, Infectious Diseases Department, Hospital Universitario Gregorio Marañón, Madrid, Spain; 7 Networking Research Center on Bioengineering, Biomaterials and Nanomedicine (CIBER-BBN), Madrid, Spain; Cornell University Joan and Sanford I Weill Medical College, UNITED STATES

## Abstract

People living with HIV-1 experience an accelerated aging due to the persistent and chronic activation of the immune system. This phenomenon conduces to immune exhaustion and precipitate immunosenescence. In general, frailty is defined as a syndrome of physiological degeneration in the elderly. Circulating naïve and memory T cells were studied by flow cytometry in non-frail and frail HIV-1-infected groups. Thymopoiesis, cell activation, senescence and cell proliferation were analyzed by CD31, HLA-DR/CD38, CD28/CD57 and Ki-67 expression, respectively. Plasma levels of sCD14 and MDA were measured by ELISA. Frail infected individuals showed a reduced number of memory T cells, both CD4^+^ and CD8^+^ populations. Activated CD3^+^CD4^+^HLA-DR^+^ T cells were lower in frail individuals, and directly correlated with CD3^+^CD8^+^HLA-DR^+^ and CD8_M_ cells. Senescent CD8^+^CD28^−^CD57^+^ cells were reduced in frail HIV-1 infected individuals and inversely correlated with CD8_RTE_, CD8_N_ and CD3^+^CD4^+^HLA-DR^+^. Higher plasma levels of sCD14 and MDA were found in HIV-1 infected frail individuals. Our data show association among frailty, markers of immune activation and oxidative stress. Understanding the immune mechanisms underlying frailty status in HIV-1 population is of high relevance not only for the prediction of continuing longevity but also for the identification of potential strategies for the elderly.

## Introduction

With the advent of antiretroviral therapy combination (ARTc), people living with HIV-1 (PLWH) are surviving to older age [1]. HIV-1 infection and aging are associated with immune activation and inflammation [2], and even with the suppression of the viral load and a partial immune reconstitution, HIV-1^+^ individuals present a profound immune activation. This activation in PLWH could contribute to aging and to the development of chronic diseases. Frailty is a common clinical syndrome in the elderly. This syndrome is related to a major susceptibility to stressors by the restricted physiological reserve [3]. A frailty phenotype was described as deterioration in three or more of five domains: physical slowness, fatigue, low activity, weakness, and physical shrinking [4]. Frailty occurs more frequently in PLWH than in HIV-1- non-infected individuals [5, 6].

Untreated HIV-1 infection results in a depletion of the naïve T cell compartment, and other perturbations in T cell homeostasis, with consequent functional impairments in the continuing cells. However, most HIV-1 individuals on ARTc experience a recovery of circulating CD4^+^ T cells [7–9]. CD4^+^ T helper (Th) cells play a great role in adaptive immune responses. Maintaining equilibrium in the Th-cell compartment is complex due to the constant turnover of the immune system and the high demands for lymphocyte replenishment [10]. A strong thymopoiesis is the key factor for an effective immunity against different infections. However, not all HIV-1 individuals with undetectable viral levels recover CD4^+^ T cell numbers. To recover the management of the CD4^+^ T cells, assessment of thymic function, peripheral T cell homeostasis and T cell loss in each infected individual is required. The thymus is the site of T cell development, producing different types of T cells that later enter the bloodstream and colonize peripheral tissues. CD31 or PECAM-1 or glycoprotein IIa, is expressed on T cells, monocytes, endothelial cells, platelets, and granulocytes. CD31 expression on CD4^+^ T cells defines recent thymic emigrants [11, 12]. The naïve CD45RA^+^ T cells that co-expressing CD31 have a higher T cell receptor excision circles (TREC) content (on average eight times higher) that identify recent thymic emigrants than CD45RA^+^ cells without CD31 expression. However, whether CD31-expressing CD8^+^ T cells are also recent thymic emigrants in healthy individuals or in HIV-1 individuals has not yet been sufficiently researched.

Immunosenescence is robustly connected with higher susceptibility to severe infections such as cancer, vaccine failure and autoimmune diseases in the elderly (reviewed by [13]). It is characterized by a progressive decrease in the absolute number of both CD4^+^ and CD8^+^ T cells. There is a decrease in the naïve cell populations and an increase in the number of dysfunctional memory cells. The senescent phenotype, in CD4^+^ and CD8^+^ subsets, is typically characterized by clonal expansion of CD28^−^ T lymphocytes. CD28 is a notable co-stimulatory molecule for T cell activation and a proliferation marker. The loss of CD28 marker is also shown to be associated with higher CD57 expression on T cells, being a marker for replicative senescence [14].

Immune activation and chronic inflammation play a very important role in the pathogenesis of age-related morbidity in PLWH and healthy populations. In comparison with paired non-HIV-infected individuals, HIV-1^+^ patients under ARTc and with virologically suppressed HIV-1 infection have an earlier occurrence of frailty and other common age-related symptoms [2].

HIV-1 infection is associated with signs of innate immune system activation. Even under an effective ARTc, levels of sCD14 remain increased and have been proposed as a potential biomarker for non-AIDS mortality risk in PLWH [15, 16]. Several lines of evidence support that HIV-1 infection is associated with oxidative stress, and this phenomenon contributes to the pathogenesis of the HIV-1 infection [17, 18].

Increased level of oxygen metabolites is an unusual phenomenon occurring inside immune cells or tissues that damages essential cellular components, resulting in abnormal gene expression, immunity perturbation, disturbance in receptor activity, mutagenesis, protein or lipofushin deposition. Reactive oxygen species (ROS) generation is closely involved in the induction of cellular senescence, and inflammation has been previously described [19, 20]. One of the most well-described consequences of the generation of free radicals and ROS is lipid peroxidation.

Healthy adults present a 2:1 ratio of CD4/CD8, and the inversion of this index is related to signs of premature immunosenescence in HIV-1^+^ groups of different ages [21]. It has been previously shown that inadequate proportion of CD4/CD8 predict HIV infection-related clinical events in infected individuals [22, 23].

To explore the association of specific CD4^+^ T and CD8^+^ T cell subsets markers of senescence, the cellular activation, the thymic function, and the immune risk profile with frailty in the elderly living with HIV-1 was our main objective. Better understanding of the mechanisms causing frailty will help to find a prevention and efficient treatment for the individuals.

## Material and methods

### Study design and patient population

An initial cross-sectional study in two university hospitals in Madrid (Spain) was performed. Our principal objective was to evaluate the prevalence of frailty in accordance with the criteria of Fried and to analyze physical function in a cohort of elderly HIV-1^+^ individuals and their results are published [22]. From the 117 elderlys included, blood samples of only 45 HIV-1^+^ individuals were available for the study of immune activation and oxidative stress.

We recorded sociodemographic data (age, gender, education and social status) and HIV-1-infection related data: duration of the HIV-1 infection (months), duration of ARTc, risk practice for HIV-1 infection, nadir CD4^+^ T cells/μL, current CD4^+^ T cell count on ARTc, current HIV-1 RNA log^10^ copies/mL, current CD4/CD8 ratio on ARTc and Centers for Disease Control and Prevention clinical category. We also recorded coinfection with other viruses: HBV (HbsAg positive) and HCV (antibodies against HCV positive, viral load, genotype, fibrosis stage, treated or not, cure or not) and other comorbidities [hypertension, type 2 diabetes, dyslipidemia, coronary heart disease, stroke, COPD, chronic renal failure, current cancer (fewer than 5 years from the diagnosis), previous cancer (more than 5 years from the diagnosis; not active disease), depression, psychiatric disease and osteoarticular disease.

### Frailty

Frailty was determined taking into account the criteria of Fried et al. [24], namely, shrinking (unintentional weight loss of ≥4.5 kg or ≥5% of body weight during the previous year), weakness (grip strength adjusted for gender and body mass index), poor endurance and energy (self-reported exhaustion identified by two questions from the Center for Epidemiologic Studies Depression scale), slowness (based on time to walk 15 feet, adjusting for gender and standing height) and low physical activity level (<383 kcal/week in men and <270 kcal/week in women using the Minnesota Leisure Time Activity Questionnaire). HIV-1^+^ patients were considered frail when they met at least three of the five criteria, prefrail when they met one or two criteria and robust when they met no criteria as it was defined by Fried.

### Physical function

Physical function was analyzed by the Short-Form Late-Life Function and Disability Instrument (SF-LLFDI) [25, 26]. The main objective was measure of strength, gait speed and balance using the Short Physical Performance Battery (SPPB) [27].

Other geriatric syndromes were recorded: falls were evaluated by self-reporting in response to the question, “have you fallen in the past year?” The number of falls was recorded and whether the fall had consequences or not (such as a visit to the general practitioner or the emergency department). Depression was tested using the Geriatric Depression Scale Short Form (GDS-SF or Yesavage Test) [28]. Risk of malnutrition was analyzed using the Mini Nutritional Assessment Short Form (MNA-SF) [29], and by objective measurements (weight, height, and body mass index).

The Veterans Aging Cohort Study (VACS) Risk Index (VACS RI), which predicts hospitalization and all-cause mortality in uninfected and HIV-1^+^ infected individuals, was used [30]. This VACS study incorporates Age and CD4 count, viral load, renal function, hemoglobin, liver fibrosis (FIB4) and HCV co-infection routinely. The VACS Index is strongly correlated with inflammatory biomarkers [31, 32].

The Cumulative Illness Rating Scale for Geriatrics (CIRS-G) [33, 34] is an index or list based on various major diseases that is generally accepted as an established comorbidity detection instrument. The severity of chronic diseases in 14 individual body systems is rated along a five grades system with a minimum score of 0 (no problem affecting that system) up to a maximum score of 4 (extremely severe problem) according to the criteria described previously in detail by Miller et al. [34]. Theoretically, the total score of the CIRS-G varies from 0 to 56.

### T cell immunophenotyping

Peripheral blood mononuclear cells (PBMCs) were isolated from whole blood by Ficoll-Paquet density centrifugation and washed twice in RPMI 1640. We applied the same standard operating procedure for the collection and processing of samples of the Spanish HIV HGM BioBank [35, 36]. Viable PBMCs were counted by trypan blue exclusion and resuspended in 10% dimethylsulphoxide/90% heat inactivated fetal calf serum for cryopreservation in liquid nitrogen. Thawed PBMCs were washed, resuspended at 10^6^ cells/mL, and stained for surface markers. The viability of fresh PBMCs was always >95%. Lymphocytes were distinguished from monocytes by their forward and side light scatter.

Fluorescently conjugated monoclonal antibodies used to assess T cell subsets were CD3-Pacific Orange, CD4-APC-Cy7, CD8-Pacific Blue, CD45RA-FITC, CD45RO-ECD, CD27-PC5, and CD31-PE (BD Biosciences; San-Jose, CA). To analyze immune activation, PBMCs were incubated with CD3-Pacific Orange, CD4-APC, CD8-PC7, CD57-FITC, HLA-DR-PC5, CD28-PE, and CD38-Pacific Blue (BD Biosciences; San-Jose, CA). From the lymphocyte population (identified by forward and side light scatter properties), CD4^+^ and CD8^+^ T cell subsets were defined as follows: naive (CD4_N_, CD8_N_) (CD45RA^+^CD27^+^), memory (CD4_M_, CD8_M_) (CD45RO^+^CD27^+^), senescent (CD4^+^CD28^−^CD57^+^, and CD8^+^CD28^−^CD57^+^). The CD31^+^ subpopulations of naïve CD4^+^ and CD8^+^ T cells were analyzed as recent thymic emigrants (CD4_RTE_ and CD8_RTE_).

Gating strategies are presented in S1 Fig. A minimum of 100,000 events per sample was analyzed using a FACS EPICS-XL MCL (BD Biosciences) and gates were set using appropriate controls. Flow Cytometry data were analyzed using Kaluza software (Beckman Coulter).

The CD4/CD8 ratio was measured in whole blood to match with previous studies.

### PBMCs cultures and detection of proliferative cells

Thawed PBMCs were washed, resuspended at 10^6^ cells/mL in RPMI 1640 with 10% fetal calf serum and cultured with or without PHA (1μg/ml) (InvivoGen) at 37°C in 5% CO_2_ for 72 hours. Intracellular Ki-67-FITC mAb (clone B56; BD-Pharmingen) or its respective control isotype was added to PBMCs after fixation and permeabilization (Cytofix/Cytoperm Plus, BD Biosciences) of PBMCs to assess cell proliferation.

### Plasma levels of sCD14 and MDA

Enzyme-Linked Immunosorbent Assay kit was used to measure plasma levels of sCD14 (R&D Systems Europe, Ltd). Lipid peroxidation was measured by plasma MDA estimation following manufacturer instructions (TBARS Assay kit, Cayman Chemical).

### Statistical analysis

Data are presented as median (interquartile range). Comparisons between groups were performed using the non-parametric Mann-Whitney U test. Associations between continuous variables were assessed by the Sperman´s rank correlation coefficient and by simple or multivariate linear regressions analyses. Statistical analysis of the data was performed using SPSS for Windows (SPSS Inc., Chicago). Statistical significance was assigned at *p<0.05, and **p<0.01, and ***p<0.001.

### Ethics statement

The Ethics Committee of each hospital approved the study. The patients were informed of the purpose, benefits, and potential risks of the study. Written informed consent was obtained from all participants.

## Results

### Characteristics of non-frail and frail groups

Our study cohort comprised 45 HIV-1^+^ individuals older than 55 years. We measured frailty using the Fried frailty status [3]. Detailed demographic and clinical characteristics of the study individuals categorized according to frailty are shown in Table 1. We had blood sample available from ten of the eighteen frail-patients. Therefore, we matched 35 control patients (non-frail) by age, nadir CD4 T-cell count, current CD4^+^ T cells, CD4/CD8 ratio, undetectable viral load and years living with HIV-1.

**Table 1:** Detailed demographic and clinical characteristics of the study individuals categorized according to frailty.

Characteristics	Non-frail	Frail	P Value
**N**	35	10	
**Age, Median (IQR), y**	61 (56–70)	61.5 (57.75–63)	0.7221
**Sex, male**	29 (82)	7 (70)	0.3861
**Transmission (%)**			
**Homosexual**	14 (40)	2 (20)	
**Heterosexual**	10 (28)	6 (60)	
**IDU (injecting drug users)**	6 (17)	2 (20)	
**Behavioral health**			
**Smoker**	11 (31.4)	3 (30)	
**Alcohol drinks per week**	1 (3.84)	1 (10)	
**HCV infection**	10 (28.6)	2 (20)	0.7202
**HIV-related health and therapy**			
**Nadir CD4 count**			
Median (IQR), cells/μl	142 (37–300)	137 (42.75–218)	0.7744
< 200 cell/μl	23 (65.7)	7 (70)	
200–499 cell/μl	9	3	
+ 500 cell/μl	3	0	
**Current CD4+ T cells**			
Median (IQR), cells/μl	581 (394.8–797)	718 (413–859)	0.393
< 200 cell/μl	0	0	
200–400 cell/μl	7	1	
400–600 cell/μl	6	3	
600 cell/μl	16	6	
**Log VL**	4.92 (3.27–5.28)	4.6 (4.35–4.96)	0.6116
**Years of infection**	17 (12–19)	18 (8.75–22.25)	0.6122
**Years received ARTc Median (IQR)**	15 (10.25–18)	17 (6.75–19.5)	0.8822
**Age at infection**	49 (38–57)	43 (37.25–54.75)	0.3969
**CD4/CD8 ratio, Median (IQR)**	0.73 (0.49–1.2)	0.69 (0.26–1.02)	0.4411
**SPPB**	9 (8–11)	7.5 (6.75–9)	0.0027**
**VACs RI**	27 (17.75–42.25)	24 (21–34.75)	0.6941
**VACs Death**	10.2 (6.1–20.6)	8.7 (7.4–15.05)	0.6941
**SFFLFDI**	133 (126–143)	110 (103–127.8)	0.0006***
**CIRS**	5 (4–8)	8.5 (7.75–10.25)	0.0120*
**Walking speed (m/s)**	1.08 (0.8–1.3)	1.485 (1.083–1.91)	0.0376
**Severe Depression (%)**	0 (0)	2 (20)	
**Nutritional status (%)**	29 (83)	7 (70)	
Normal	5 (14)	3 (30)	
Risk	1 (3)		
Malnourished			

Statistical significance was assigned at *p<0.05, and **p<0.01, and ***p<0.001. IDU, injecting drug users; HCV, hepatitis C virus; VL, viral load; ARTc, antiretroviral therapy combination; SPPB, Short Physical Performance Battery; VACs RI, Veterans Aging Cohort Study risk index.

Most of the study participants were male (80%) with a mean age of 61 years old; The median of the current age in the non-frail group was 61 (56–70) and 61.5 (57.75–63) in the frail group.

When correlation analysis was performed, we found that frail status showed a positive correlation with depression state (r = 0.473; ***p = 0.001), nutritional status (r = 0.347, *p = 0.02), MDA levels (r = 0.341; *** *p* = 0.022) and CIRS total (r = 0.392, * *p* = 0.008). Negative association was detected between frailty status and SPPB (r = -0.604; ***p<0.001), and SFFLLDI (r = -0.71; ***p<0.001). Moreover, frailty inversely correlated with CD3^+^CD4^+^HLA-DR^+^ T cells (%) (r = -0.372; **p<0.05) (Table 2).

**Table 2:** Correlation analysis between IFried and diverse variables cited in the text.

	CIRs Total	Yesavage	MNA-SF	MDA	SPPB	CD4 HLADR	SFLLFDI
**Ifried**							
Correlation Coefficient	0.392	0.473	0.347	0.341	-0.604	-0.372	-0.71
*P*	0.008	0.001	0.02	0.022	0.001	0.014	0.001

CIRS, Cumulative Illness Rating Scale; MNA-SF, Mini Nutritional Assessment Short Form; MDA, malondialdehyde; SPPB, Short Physical Performance Battery; SF-LLFDI, Short-Form Late-Life Function and Disability Instrument.

We compared individual markers between frail and non-frail groups to determine whether there were significant statistical differences in any of the parameters described above (S2 Fig). When the current number of circulating CD4 T lymphocytes was analyzed, we found that the frail group showed higher number of CD4^+^ T cells 718 (413.8–859) *vs*. 581 (394.8–797) non-frail group in peripheral blood although without statistical significance (Table 1). It is important to consider that frail and non-frail groups were significantly different in various characteristics such as HIV-1 frail group presented heterosexual transmission at 60% *vs*. 28% in non-frail group, and 10% *vs*. 40% were men who have sex with men. We found statistical differences between both frail and non-frail groups in SF-LLFDI (*p<*0.001), CIRS total (*p = 0.012), and SPPB (**p = 0.0027) (see Table 1). However, there was no statistical difference regarding sex/gender, current age, age at infection, years of HIV-1 infection, years on ARTc, nadir, CD4/CD8 ratio or logVL between both groups. Also, no statistical differences were found in VACSRI, or walking speed. In contrast with previously reported studies [37], we did not find any difference in the rate of HCV infection between frail and non-frail groups (20% *vs*. 28.6%). A similar percent of frail and non-frail HIV-1^+^ individuals were receiving ARTc (72.3% *vs* 74.8%, respectively). It is interesting to note that in the frail group 2/10 individuals presented severe depression compared to none in the non-frail group. Also, we performed exploratory assays of the association of frailty status with the phenotype of immune cell, senescence markers and immune cell subsets activation.

### Levels of CD4 and CD8 T cells in frail individuals

**Fig 1:**
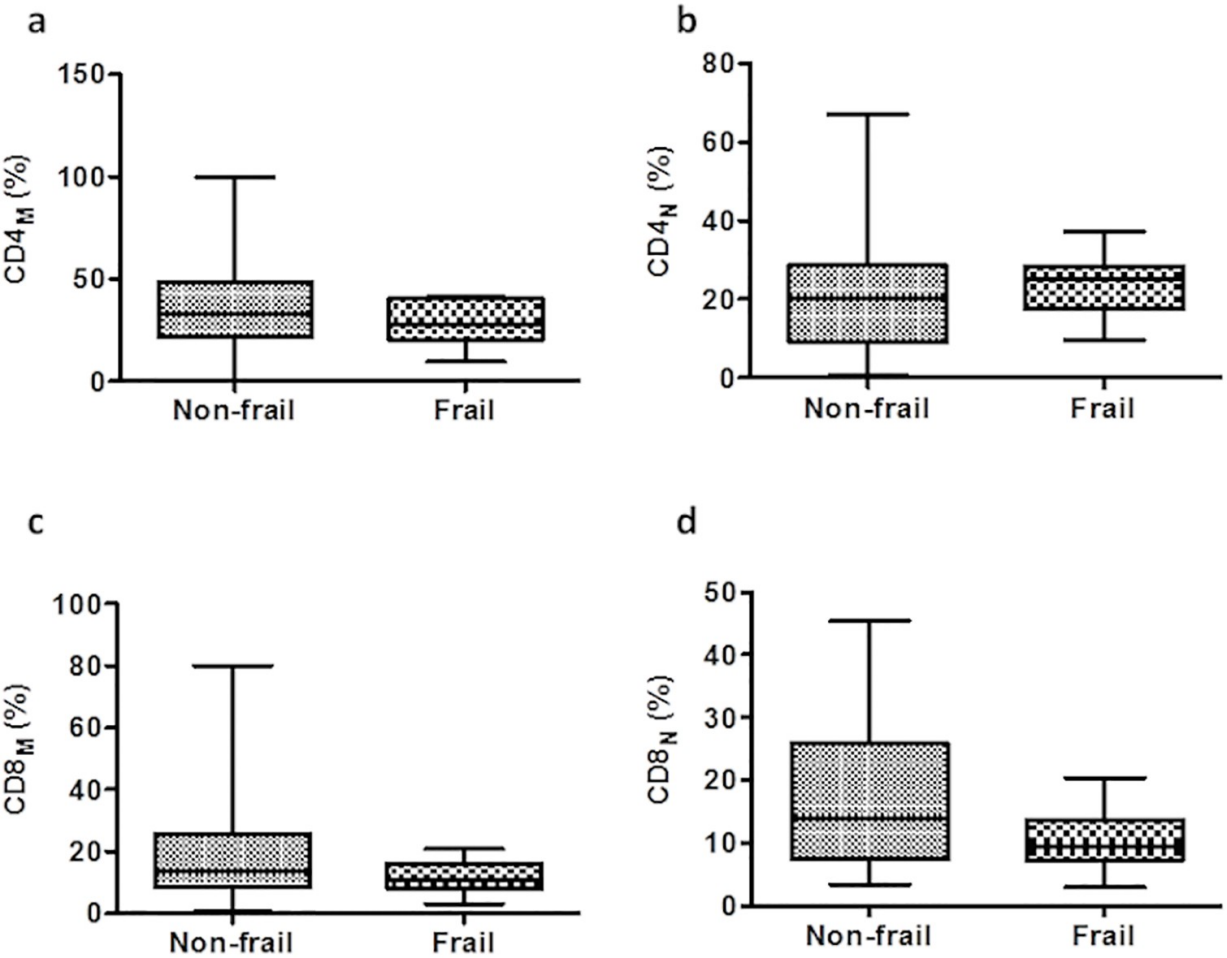
Percentages of CD4^+^ and CD8^+^ T cell subpopulations in frail and non-frail groups. PBMCs were analyzed by flow cytometry by first gating on the total mononuclear cells and then on the CD3^+^ T cells. The frequency of CD4_M_ (a), CD4_N_ (b), CD8_M_ (c), CD8_N_ (d) T cells was then determined. Box plots represent median with 25th and 75th percentile borders, error bars represent 10 th and 90 th percentile. The mean ± SEM for each group is given below the bar for that particular group.

Association neither positive nor negative was found between frailty and naïve or memory subsets. One of the strongest correlates for CD4^+^ T cell decline in HIV-1 infection is the increased frequency of CD4_M_ T cells and a corresponding deficit of CD4_N_ T cells [38]. The Frail group exhibited a decreased frequency of CD4_M_ cells (27.63 *vs*. 32.88) and an increase in CD4_N_ (25.11 *vs*. 20.05) compared to the non-frail group, but these results were not statistically significant (Fig 1a and 1b). When the CD8_M_ and CD8_N_ levels were explored, we found that the frequency of CD8_M_ and CD8_N_ subsets were reduced in the frail group compared to the non-frail group (10.94 *vs*. 13.61, and 9.47 *vs*. 13.74, respectively), but again the differences were not significant between both groups (Fig 1c and 1d).

### Cell activation

**Fig 2:**
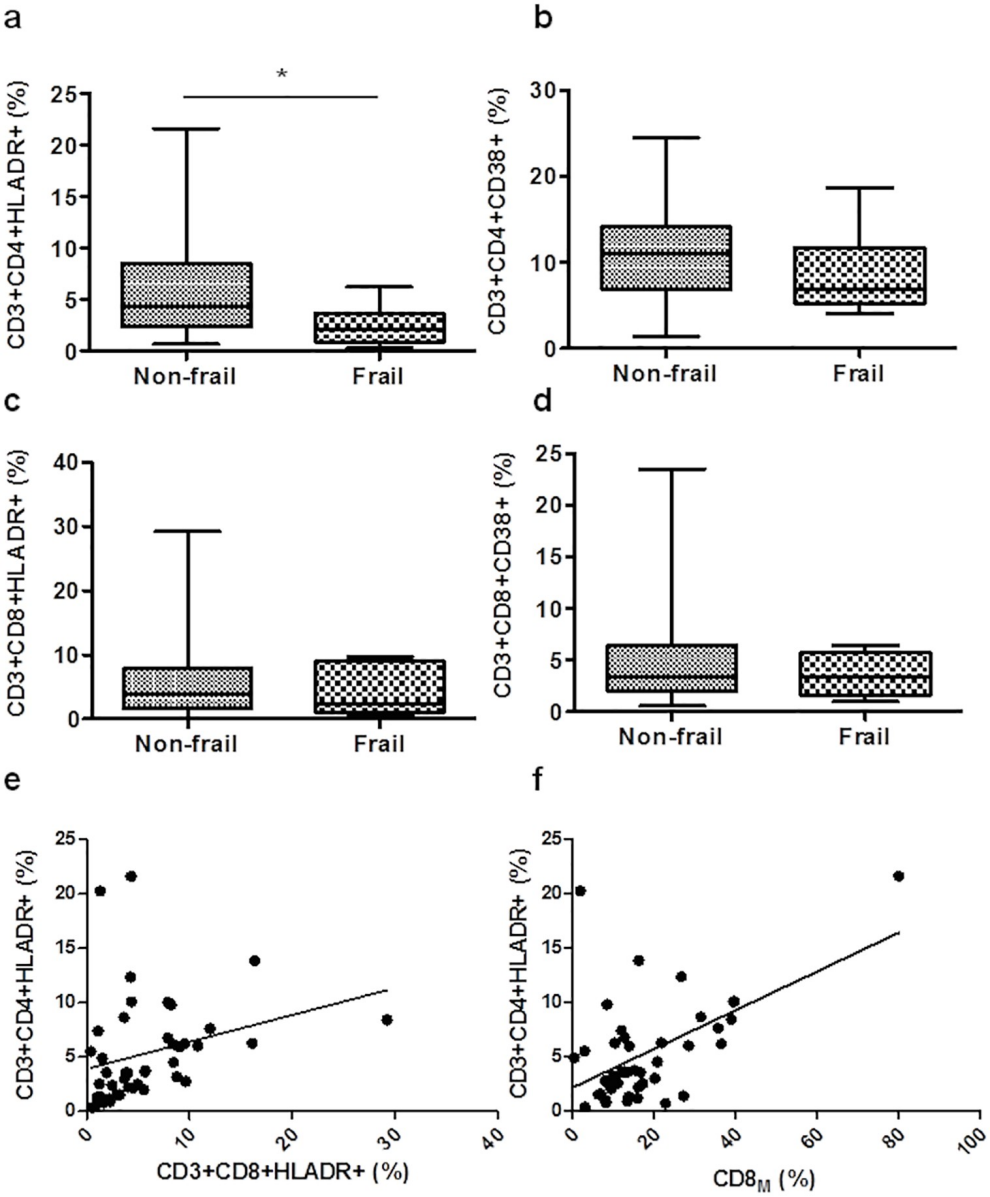
Immune activation by the measure of CD38 and HLA-DR markers was analyzed by flow cytometry in CD4^+^ and CD8^+^ T cells of frail and non-frail groups. Box plots show the frequency of activated T cells as follows: CD3^+^CD4^+^HLA-DR^+^ (a), CD3^+^CD4^+^CD38^+^ (b), CD3^+^CD8^+^HLA^−^DR^+^ (c) and CD3^+^CD8^+^CD38^+^ (d). Box plots represent median with 25th and 75th percentile borders, error bars represent 10th and 90th percentile. The mean ± SEM for each group is given below the bar for that group. Positive correlations between CD3^+^CD4+HLA-DR^+^ (%) with CD3^+^CD8+HLA-DR^+^ (e), and CD8_M_ (f). *p<0.05.

We evaluated the activation state of CD4^+^ and CD8^+^ T cells by measuring the surface presence of CD38 and HLA-DR. We found, in the peripheral cells, that a reduction in the frequency of the CD3^+^CD4^+^HLA-DR^+^ T cells was identified as a frailty-associated phenotype (r = -0.372, *p = 0.014). Frail group had lower frequencies of both CD3^+^CD4^+^HLA-DR^+^ (*p<0.05) and CD3^+^CD4^+^CD38^+^ subpopulations than non-frail group but in the last case this frequency was not statistically significant (Fig 2a and 2b, respectively). Among CD3^+^CD8^+^ cells, the cellular activation measured as CD3^+^CD8^+^HLA-DR^+^ (%) and CD3^+^CD8^+^CD38^+^ (%) did not differ significantly by frailty status (Fig 2c and 2d). The proportion of total CD4^+^ T cells expressing HLA-DR correlated directly with CD3^+^CD8^+^HLA-DR^+^ (%) (r = 0.511, ***p<0.001) (Fig 2e), and with CD8_M_ (%) (r = 0.388, *p = 0.009) (Fig 2f).

### Increased frequency of CD8 senescent cells in the frail group

**Fig 3:**
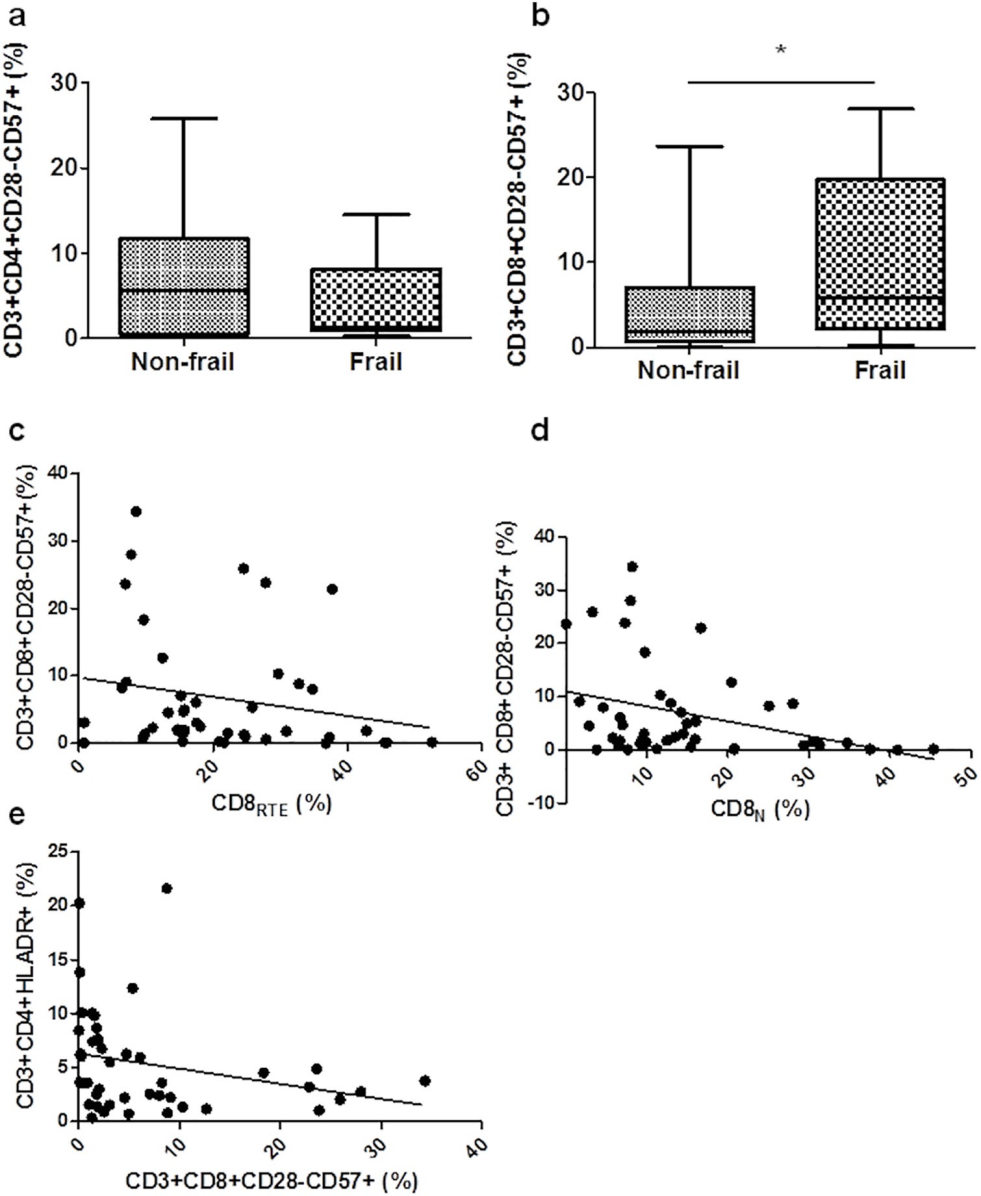
Increased frequency of CD57-expressing CD3^+^CD8^+^ T cells in HIV-1^+^ frail group. PBMCs were assayed by flow cytometry. The frequencies of CD57^+^ cells among the CD3^+^CD4^+^CD28^−^ (a) and CD3^+^CD8^+^CD28^−^ (b) T cells were determined. Box plots represent median with 25th and 75th percentile borders, error bars represent 10th and 90th percentile. The mean ± SEM for each group is given below the bar for that group. Negative associations between CD3^+^CD8^+^CD28^−^CD57^+^ (%) and CD8_RTE_ (c), CD8_N_ (%) (d), and CD3^+^CD4^+^HLA-DR^+^ (%) (e). *p<0.05.

The loss of CD28 and the increased expression of CD57 defines the T cell senescence in both CD4 and CD8 T cells. When we explored the expression of these markers in CD3^+^ T cells, we observed that the frequency of CD4^+^CD28^−^CD57^+^ T cells did not differ significantly between both groups (Fig 3a), whereas in the frail group the frequency of CD8^+^CD28^−^CD57^+^ was significantly elevated in comparison with the non-frail group (*p = 0.017) (Fig 3b).

When the total frail and non-frail groups were analyzed, CD8^+^CD28^−^CD57^+^ (%) inversely correlated with CD8_RTE_ (r = -0.300, *p = 0.048) (Fig 3c), CD8_N_ (%) (r = -0.355, *p = 0.017), (Fig 3d), and CD3^+^CD4^+^HLA-DR^+^ (%) (r = -0.415, **p = 0.005) (Fig 3e).

### Impaired CD31 expression in CD8^+^CD45^+^ T cells in the frail group

**Fig 4:**
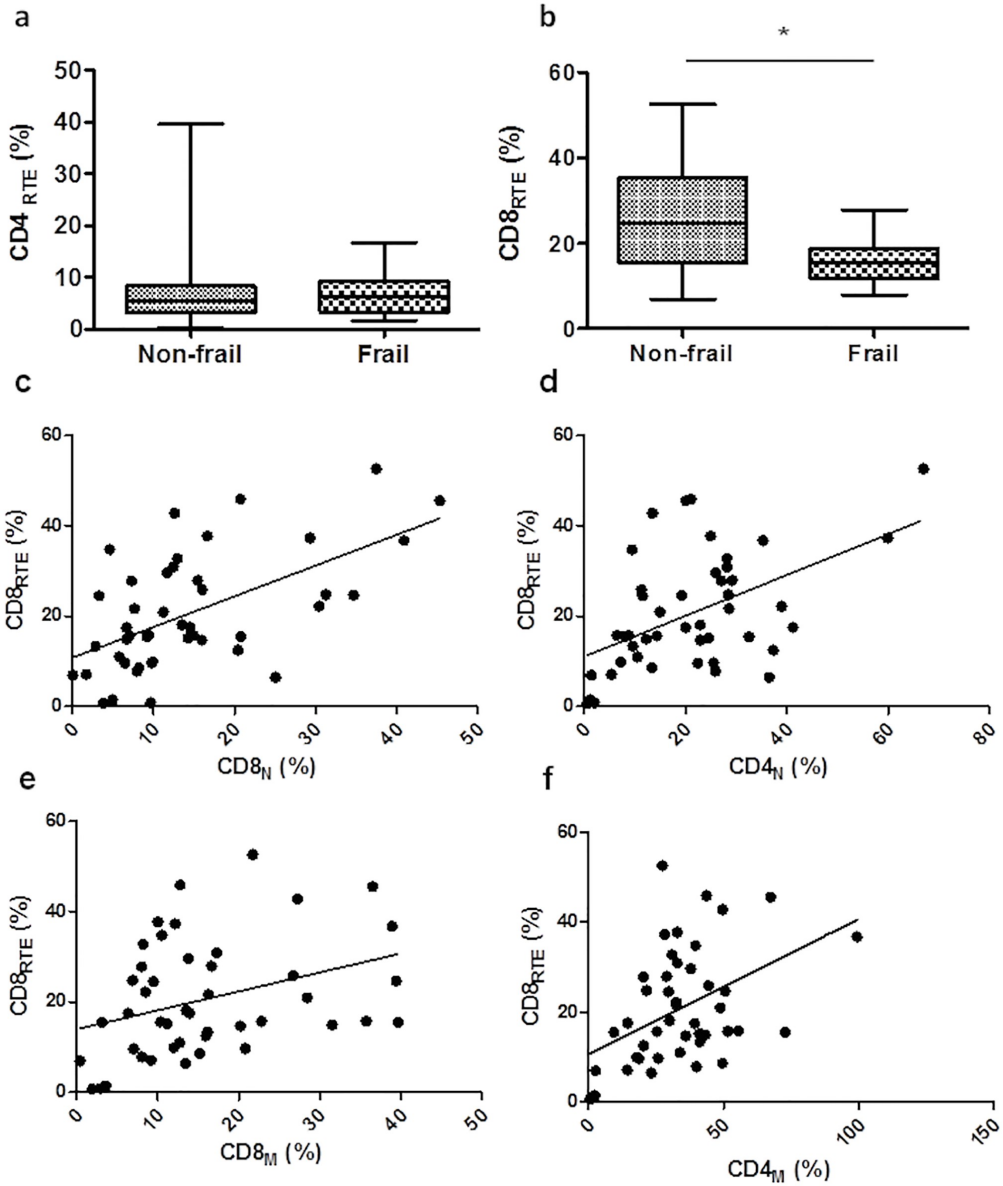
Reduction of the CD8_RTE_ cells in frail compared to non-frail groups. PBMCs were assayed by flow cytometry by first gating on the total mononuclear cells and then on the CD3^+^ T cells. The frequencies of CD4_RTE_ (a), and CD8_RTE_ (b) were then determined. Box plots represent median with 25th and 75th percentile borders, error bars represent 10th and 90th percentile. The mean ± SEM for each group is given below the bar for that particular group. Positive correlations between CD8_RTE_ (%) and CD8_N_ (%) (c), CD4_N_ (%) (d), CD8_M_ (%) (e), and CD4_M_ (%) (f). *p<0.05.

Although few studies have assessed the expression of CD31 on CD8^+^ T cells as a marker of recent thymic origin, due to the low proportions of naïve CD8^+^ T cells, we wondered if a failure in the generation of new Th cells was occurring in the frail group. Specifically, we measured the presence or absence of the CD31 surface marker expression as a marker of undergoing proliferation in the periphery. The percentage of CD4^+^ T cells co-expressing CD45 and CD31 was similar [median 5.3 (3.2–8.4) *vs*. 6.33 (3.34–9.35)] between both groups but did not reach significance (Fig 4a). The frail group exhibited lower CD8_RTE_ T cells counts compared to the non-frail group (*p* = 0.02) (Fig 4b). Positive correlations of CD8_RTE_ (%) were found with CD8_N_ (%) (r = 0.536, ***p<0.001) (Fig 4c), CD4_N_ (%) (r = 0.451, **p = 0.002) (Fig 4d), CD8_M_ (%) (r = 0.364, *p = 0.014) (Fig 4e), and CD4_M_ (%) (r = 0.435, **p = 0.003), (Fig 4f). CD8_RTE_ in HIV-1 individuals may be diluted by mature CD8^+^ T cells that also express CD31. In this study, we have not addressed this issue. However, it would be useful to assess the expression of CD57 and CCR7 on CD4^+^ and CD8^+^ T cell subsets that co-expressed CD45RA and CD31.

### Frailty is not associated with changes in peripheral proliferation of T cells

**Fig 5:**
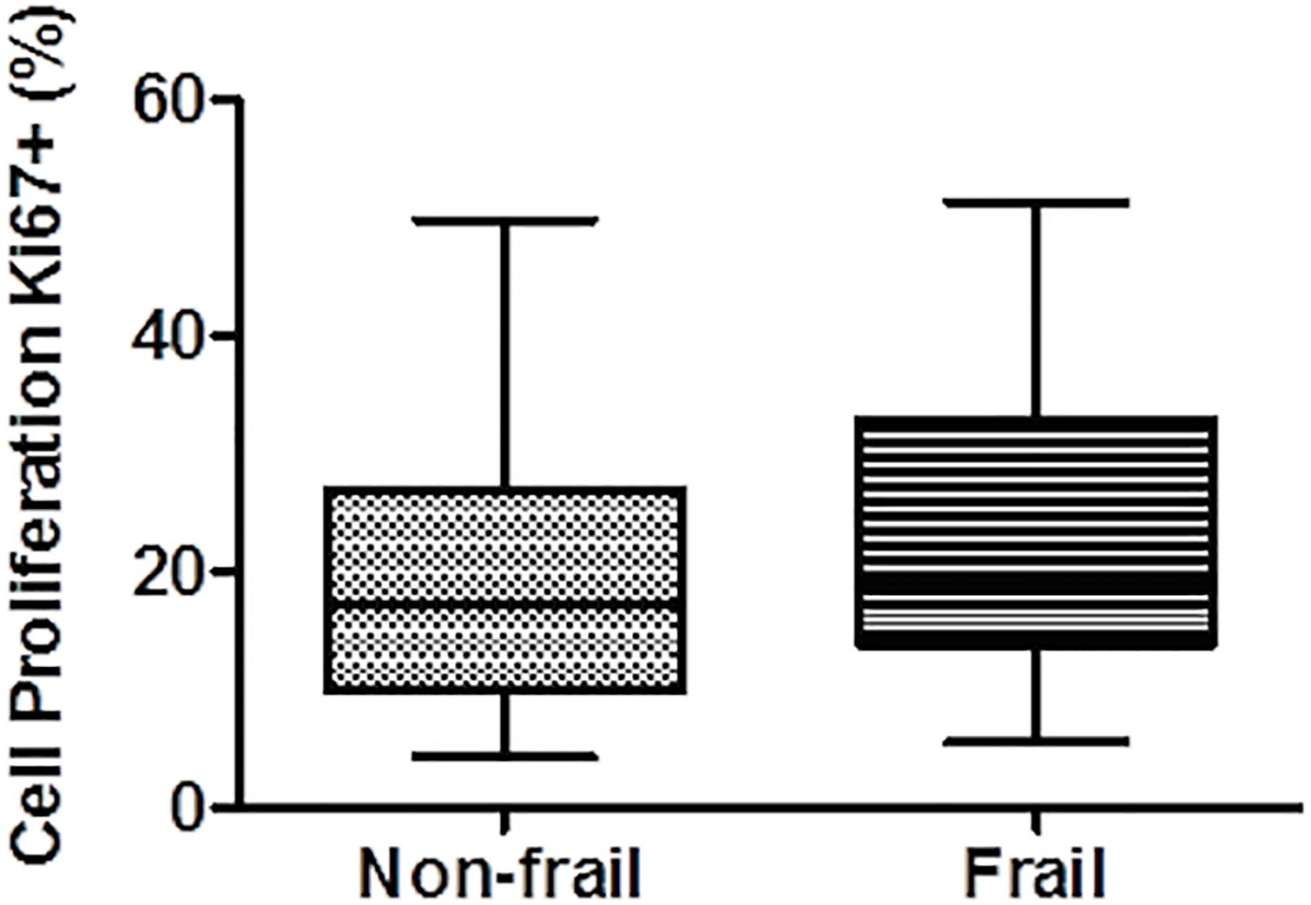
Cell proliferation of T lymphocytes in frail and non-frail groups. PBMCs isolated from HIV-1^+^ individuals were permeabilized and stained with anti-CD3, and anti-Ki-67 antibodies with or without stimulation during 3 days with PHA (1μg/ml). Representative flow cytometry profiles showing Ki-67 expression in both groups. Box plots represent median with 25th and 75th percentile borders, error bars represent 10th and 90th percentile. The mean ± SEM for each group is given below the bar for that particular group.

We measured peripheral proliferation in T cells by means of the nuclear protein Ki-67, an intracellular marker expressed during all active phases of cell division [39]. We observed a slightly increased level of proliferation in frail *vs*. non-frail groups (median 23.48 *vs*. 18.82), but the result was not statistically significant (Fig 5). This higher activation of T cells in the frail group could be explained as a homeostatic process to compensate for the decreased number of the most T cells subpopulations.

### Plasma levels of immune activation markers

**Fig 6:**
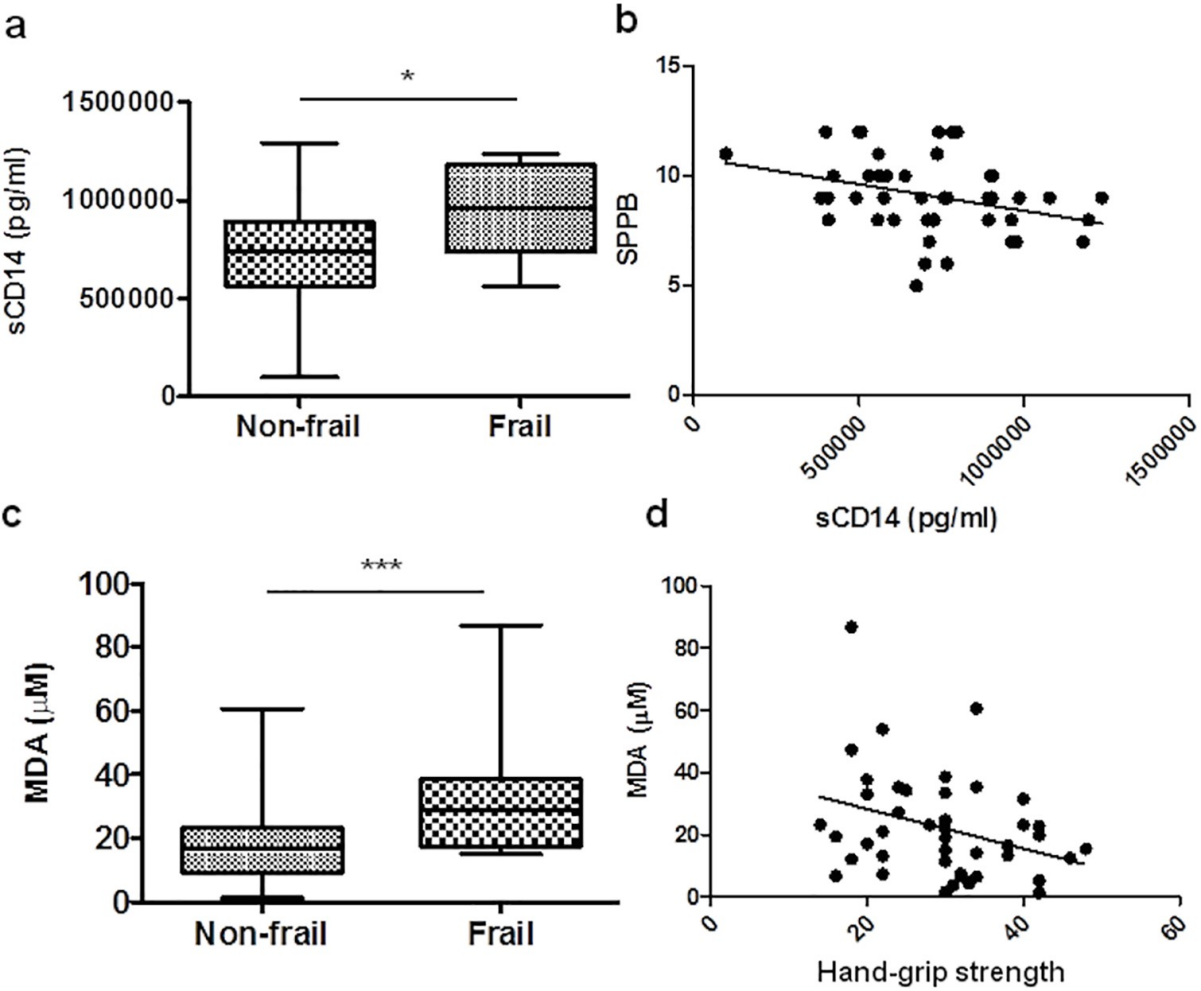
Plasma levels of inflammation biomarkers measured by ELISA. sCD14 levels (pg/ml) (a), and MDA (μM) (c) production were measured in frail and non-frail groups. Box plots represent median with 25th and 75th percentile borders, error bars represent 10th and 90th percentile. The mean ± SEM for each group is given below the bar for that group. Inverse correlation of sCD14 levels with SPPB (b), and MDA levels with strength measured by hand-grip strength as one of the components of frailty phenotype (d). *p<0.05, ***p<0.001.

During the chronic phase of HIV-1 infection, higher sCD14 plasma levels are associated with immune activation, intestinal dysbiosis and microbial translocation [40]. In this study, compared to the non-frail group, the frail group presented significantly elevated levels of sCD14 (median 939.397 pg/mL *vs*. 734.431 pg/mL, *p = 0.016) (Fig 6a). Moreover, sCD14 levels were associated inversely with SPPB (r = -0.329, *p = 0.027) (Fig 6b).

Lipid peroxidation is a chain reaction started by free radicals attacking fatty acid side chains in the phospholipids of cell membranes. A common method to evaluate the extent of oxidative stress *in vivo* is to measure lipid peroxidation product such as malondialdehyde (MDA). To measure lipid peroxidation product, such as MDA, an oxidative stress *in vivo* has been used. Compared to the non-frail group, frail group had significantly higher levels of MDA (**p = 0.0033) (Fig 6c), suggesting lipid peroxidation. Levels of free radicals or ROS can inflict direct damage to lipids as it has been previously showed (reviewed by [41]). It could be considered that the oxidative stress in frailty individuals can play a special role. In this regard, frail group was likely to present higher levels of MDA (r = 0.341, *p = 0.022), and in the total of individuals, MDA levels were inversely correlated with strength measured by hand-grip strength as one of the components of frailty phenotype (r = -0.302, *p = 0.044).

## Discussion

Our comprehensive study was performed to assess immune function in the frail HIV-1 elderly group compared to non-frail group (pre-frail and robust).

Cross-sectional studies have linked frailty in PLWH with multiple factors. Some of these markers are lower current CD4 T^+^ cell count (measured continuously [5], lower nadir CD4 T^+^ cell count [42], as well as HCV coinfection [37], low body mass index (BMI) [43], high BMI [44], lipodystrophy [44], or depressive symptoms [43]. Depression can be a consequence or a contributor to frailty [45]. In accordance with this, a positive association was found between depression status and frailty (Table 2). In addition, depression was more common in the frail group measured by the GDS-SF (2/10) compared to the non-frail group where none of the individuals was classified within the group of deep depressions and only 6 in 35 individuals showed a moderate depression.

Frailty in HIV-1 infection highly correlates with a high VL and a decline in CD4^+^ T cells [46], and the CD4/CD8 ratio has been suggested as a marker of individuals at risk of suffering from non-AIDS-related events [23]. In our study, we did not find any relationship between the level of frailty and duration of HIV-1 infection, VL, nadir of CD4, current CD4, time on ARTc in accordance with the study of Kooij et al [47]. Moreover, no differences between both groups in any of the cited parameters were found.

When positive or negative associations were studied between frailty phenotype and some immune cell populations, only a negative association was found with CD4^+^HLA-DR^+^ cells. HIV-1 infection alters the immune homeostatic mechanisms that control T cell subsets leading to progressive loss of the pool of naïve and memory T cell. In the frail group, the CD8^+^ T cells, both naïve and memory, are mainly diminished, although without reaching significant differences.

Wallet *et al*. published that greater frequency of CD4^+^CD45RO^+^ memory T cells was associated with a drop in the CD4^+^ T cell population [48]. Even with successful ART, immune activation is persistent, and it is characteristic of chronic HIV-1 infection. This phenomenon leads to the development of non–AIDS-defining co-morbidities [49].

Although previous studies showed that HIV-1 infection and aging were linked with T cell activation [50], more recent studies proved that HIV-1 infection, but not frailty, was linked to significantly higher immunosenescence and immune activation [51]. T cell activation and senescence markers are known to be higher in HIV-1^+^ infected than among HIV-1^+^ uninfected individuals, but how much this change is attributable to frailty or to HIV-1 infection is unclear. In our study, frail status was negatively associated with CD3^+^CD4^+^HLA-DR^+^ (%), and no differences were observed in activation status (CD38 and HLA-DR overexpression) between frail and non-frail groups. Our results are similar to other works which describe that T cell activation is not a predictor for serious non-AIDS events development [52, 53].

It has been reported that CD8^+^ T cells express CD38 and HLA-DR at various, but partially overlapping development and maturation stages. CD38 is expressed in immature CD8_N_ T cells and activated memory T cells, and HLA-DR is exclusively expressed on activated CD8^+^ T cells related to chronic viral infection [54, 55]. In this research, none of the markers reached the significance threshold of *p*<0.05 in any of both frail and non-frail groups. HLA-DR expression has been negatively correlated with TREC content of PBMCs [56]. Although we did not measure TREC content, we did not find any correlation among CD8^+^HLA-DR^+^ (%) and CD4^+^_RTE_ (%) or CD8^+^_RTE_ (%). In this study, thymic function measured as CD4^+^_RTE_ (%), CD8^+^_RTE_ (%) depended on immune activation measured as CD8^+^CD38^+^ (%), but not when measured as CD8^+^HLA-DR^+^ (%). An increased population of CD57 expressing CD8^+^ T cells has been shown in both aging [57] and HIV-1 infection [14]. The expansion of CD57-expressing T cells is a marker of the advancing age and HIV-1 infection [58]. Although the negative correlation between CD8^+^CD28^−^CD27^+^ and frailty (*p* = 0.068) is not statistically significant, the idea that the maintenance of a grate subsets of naïve and early memory cells is linked to a delay in the development of frailty in elderly. Increases of CD8^+^CD28^−^ T cells are observed in chronic viral infections, such us HCV, HCMV or EBV as well as in most individuals with normal aging, whereas CD4^+^CD28^−^ T cells are infrequent in most elderly individuals. [59].

The predominance of HIV-specific cytotoxic T cells lacking CD28 expression is related with progression of the disease [60] or AIDS development [61]. However, other studies have suggested that CD28/HLA-DR expression on CD4^+^ T cells, but not on CD8^+^ T cells, is an important predictor for progression to AIDS [62]. Our results clearly suggest a causal relationship between the loss of CD28 expression from CD8^+^ T cells and a frailty phenotype. CD28 is a co-stimulatory molecule on T lymphocytes, which upon binding to CD80 on Ag-presenting cells, promote cellular survival and proliferation by inducing expression of IL-2 and its receptor, stabilizing several cytokine mRNAs and activating the telomerase among other things [63].

The lack of association with CD57 suggests that even if frailty and immunosenescence occur in parallel, but some pathways are probably independent and still unknown. With the objective to establish this ‘immune frailty’ as a potential prognostic index of adverse health outcomes further studies should be performed. The association between inflammation and frailty is consistent with previous reports in HIV-1^+^ individuals, regardless of frailty definition, range of age, or HIV-1^+^ risk factors. Markers of inflammation have been associated with frailty in the general population as well as in HIV-1^+^ individuals [64, 65]. Also, we investigated the role of inflammation in frailty. We verified HIV-1^+^ frail individuals to have elevated levels of sCD14 and MDA as markers of inflammation and the last one was associated with frailty. We have not studied monocyte phenotypic alterations in HIV-1^+^ individuals, despite notable high plasma levels of sCD14. Therefore, we cannot confirm that the frail group presents a decreased frequency of peripheral blood monocytes in comparison with the non-frail group. The main limitation of all analyses performed in our study is the restriction of the sample size. Therefore, we were not able to include an HIV-1 uninfected frail control group. We propose in this report, multiple potential predictors of frailty that should now be examined in larger, longitudinal studies. Observation of alterations in several of the biomarkers previous to the onset of frailty could supply a basis for future pre-interventions and for the analysis of the efficacy of anti-inflammatory treatments.

In summary, our results clearly demonstrate associations among frailty, markers of immune activation and oxidative stress. The routine determination of frailty and physical status in older PLWH could increase the early identification of persons who could restore their functional condition by an early intervention. It is necessary the collaboration between different clinical specialists to diagnose these persons.

## Supporting information

S1 Fig Gating strategy for the identification of T cell subpopulations by flow cytometry. Forward and side scatter gates were used to mark cells with characteristics of lymphocytes and were gated by expression of CD3 (a), and CD4 cells were defined as CD3^+^CD4^+^ and CD8 as CD3^+^CD8^+^. Phenotypes of T cell subpopulation were determined using CD45RA, CD45RO, CD28, CD57, CD31 mAbs as follows: CD45RA (naïve) CD45RO (memory), CD28^−^CD57^+^ (senescent) CD31 (recent thymic emigrants).
(TIF)

S2 Fig Differences between SFLLFDI, CIRS total and SPPB in frail and non-frail groups. Box plots represent median with 25th and 75th percentile borders, error bars represent 10th and 90th percentile. The mean ± SEM for each group is given below the bar for that group. *p<0.05, **p<0.01, ***p<0.001.
(TIF)
