# Supplementary figures and images for: Frailty, markers of immune activation and oxidative stress in HIV infected elderly
Source: PLoS One. 2020 Mar 18;15(3):e0230339. doi: 10.1371/journal.pone.0230339 (PMC7080240; doi:10.1371/journal.pone.0230339)

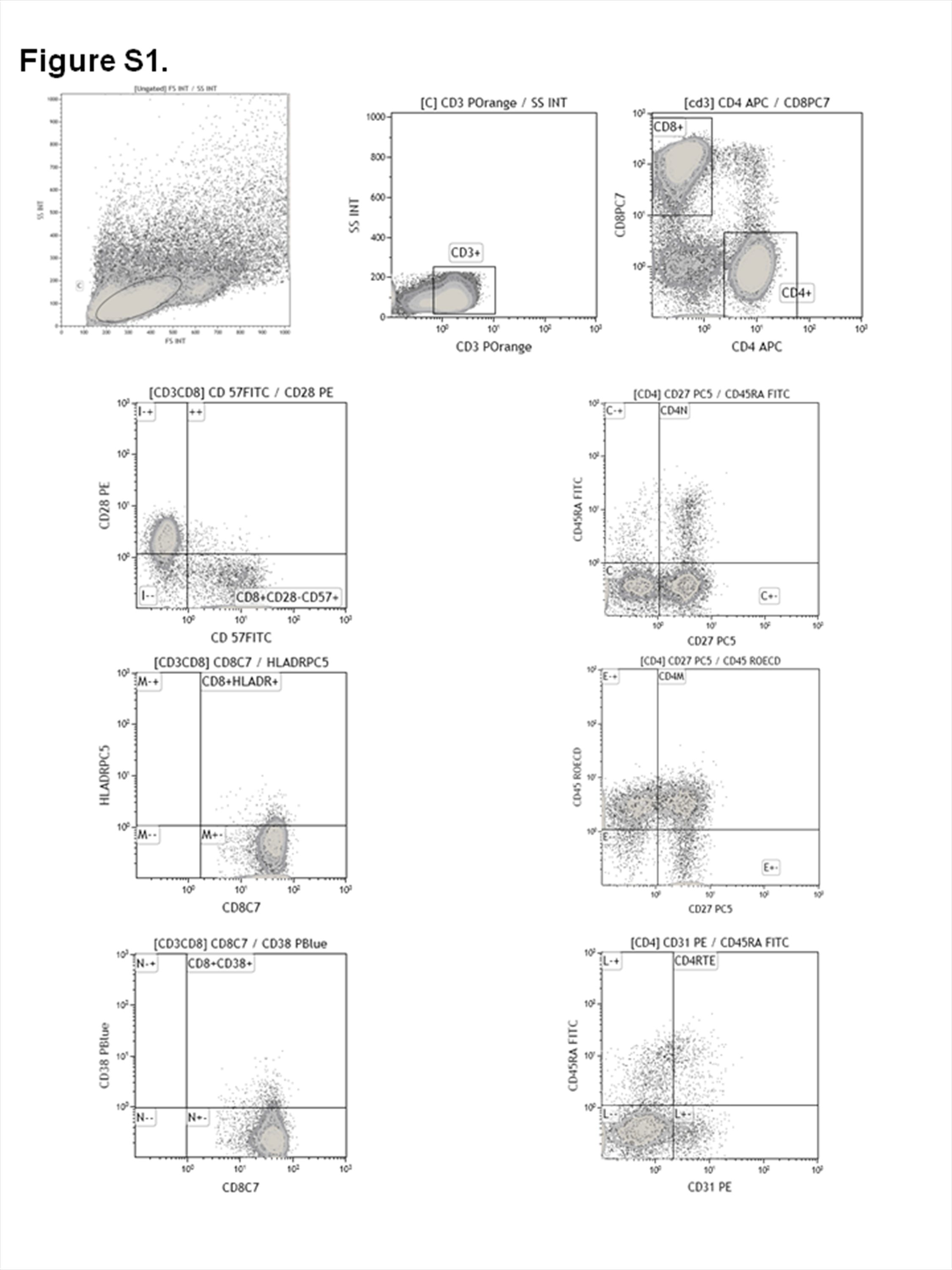

Supplement: S1 Fig — Forward and side scatter gates were used to mark cells with characteristics of lymphocytes and were gated by expression of CD3 (a), and CD4 cells were defined as CD3+CD4+ and CD8 as CD3+CD8+. Phenotypes of T cell subpopulation were determined using CD45RA, CD45RO, CD28, CD57, CD31 mAbs as follows: CD45RA (naïve) CD45RO (memory), CD28−CD57+ (senescent) CD31 (recent thymic emigrants). (TIF) [file pone.0230339.s001.TIF]

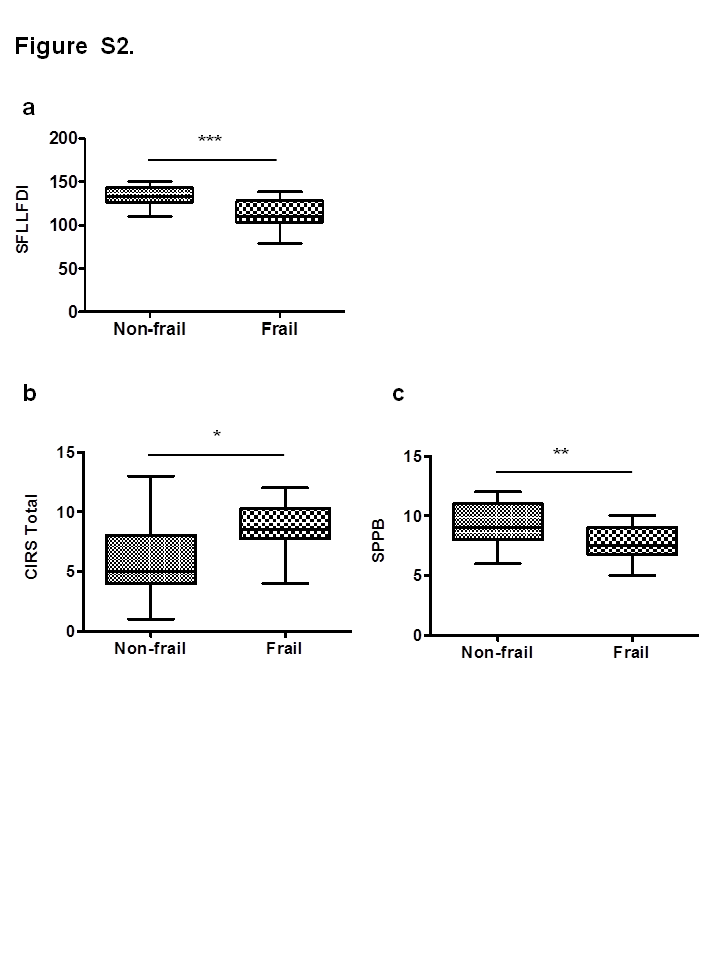

Supplement: S2 Fig — Box plots represent median with 25th and 75th percentile borders, error bars represent 10th and 90th percentile. The mean ± SEM for each group is given below the bar for that group. *p<0.05, **p<0.01, ***p<0.001. (TIF) [file pone.0230339.s002.TIF]
